# Evolutionary history of the cytochrome P450s from *Colletotrichum* species and prediction of their putative functional roles during host-pathogen interactions

**DOI:** 10.1186/s12864-023-09858-5

**Published:** 2024-01-12

**Authors:** Jossue Ortiz-Álvarez, Sioly Becerra, Riccardo Baroncelli, César Hernández-Rodríguez, Serenella A. Sukno, Michael R. Thon

**Affiliations:** 1 Institute for Agrobiotechnology Research (CIALE), Department of Microbiology and Genetics, University of Salamanca, Villamayor, Salamanca Spain; 2 Present Address: Programa “Investigadoras e Investigadores por México” Consejo Nacional de Humanidades, Ciencias y Tecnologías (CONAHCyT), Mexico City, México; 3 Department of Agricultural and Food Sciences, University of Bologna, Bologna, Italy; 4 Laboratorio de Biología Molecular de Bacterias y Levaduras, Departamento de Microbiología, Escuela Nacional de Ciencias Biológicas, Instituto Politécnico Nacional, Ciudad de Mexico, México

**Keywords:** *Colletotrichum* Graminicola species complex, P450 enzymes, CYPome, Evolution, Plant-pathogenic fungi

## Abstract

The genomes of species belonging to the genus *Colletotrichum* harbor a substantial number of cytochrome P450 monooxygenases (CYPs) encoded by a broad diversity of gene families. However, the biological role of their CYP complement (CYPome) has not been elucidated. Here, we investigated the putative evolutionary scenarios that occurred during the evolution of the CYPome belonging to the *Colletotrichum* Graminicola species complex (s.c.) and their biological implications. The study revealed that most of the CYPome gene families belonging to the Graminicola s.c. experienced gene contractions. The reductive evolution resulted in species restricted CYPs are predominant in each CYPome of members from the Graminicola s.c., whereas only 18 families are absolutely conserved among these species. However, members of CYP families displayed a notably different phylogenetic relationship at the tertiary structure level, suggesting a putative convergent evolution scenario. Most of the CYP enzymes of the Graminicola s.c. share redundant functions in secondary metabolite biosynthesis and xenobiotic metabolism. Hence, this current work suggests that the presence of a broad CYPome in the genus *Colletotrichum* plays a critical role in the optimization of the colonization capability and virulence.

**Supplementary Information:**

The online version contains supplementary material available at 10.1186/s12864-023-09858-5.

## Background

Species belonging to the genus *Colletotrichum* are plant-pathogen agents that cause anthracnose disease in many crops around the world [1, 2]. Thus, *Colletotrichum* is included as one of the most important plant-pathogenic fungi, because these species may provoke losses up to 100% in many food crops, generating serious economic repercussions [3–5]. In particular, the Graminicola species complex (Graminicola s. c.) comprehends a well-defined monophyletic clade comprised of host-specific species [6]. This complex is shaped by plant pathogenic fungal species such as *Colletotrichum graminicola* on maize, *Colletotrichum falcatum* on sugarcane, *Colletotrichum sublineola* on sorghum and *Colletotrichum eremochloae* on cultivated turfgrasses [1, 6–8].

Many *Colletotrichum* species exhibit a multistage hemibiotrophic infection strategy employing a set of several mechanical and enzymatic mechanisms during plant invasion [2, 9, 10] including Carbohydrate-Active Enzymes (CAZymes), proteolytic enzymes, necrosis and ethylene-inducing peptide 1 (Nep1)-like proteins (NLPs), effector protein candidates and secondary metabolites with phytotoxic activity, among others [11–13]. These virulence factors are important for fungal penetration and development in the host tissues, suppression of the host immune system, as well as cell and tissue destruction [2, 14, 15].

The superfamily of cytochrome P450 enzymes (CYPs) are multi-extended heme-thiolate enzymes that are found in all biological domains [16, 17]. The cytochrome P450 enzymes (CYPs), participate in cellular processes such as biosynthesis of secondary metabolites, utilization of compounds as sole carbon and energy sources, and cellular detoxification, among others [18–23]. CYPs are particularly widely expanded among filamentous fungi, conferring the capability to live in diverse habitats [24, 25].

Plant-pathogenic fungi commonly possess a great diversity of CYPs classified in multiple protein families. In fact, plant pathogenic fungi often possess larger numbers and diversity of CYPs than other fungi. For instance, *Magnaporthe oryzae*, *Cryphonectria parasitica*, *Aspergillus flavus*, *Botrytis cinerea* and *Grosmannia clavigera* each harbor more than 50 CYPs [26]. In general, fungal CYPs fulfill several functions during plant-host interaction, having important roles during fungal development and virulence [27, 28]. Numerous CYP families play parts in mycotoxin biosynthesis pathways [29–32]. Also, CYPs are implied in conidia germination and have critical roles in the ergosterol and hormone-like biosynthesis pathways during cell growth and reproduction [33–35]. Additionally, CYPs confer cell detoxification against antimicrobial compounds secreted by the plant host [19, 36, 37].

Some members of the genus *Colletotrichum* possess a large arsenal of *CYP* genes encoding P450 enzymes, comprising in some cases approximately 1% of all gene content encoded in their genomes [38]. For instance, more than 200 *CYP* genes have been recognized in *Colletotrichum higginsianum* and *Colletotrichum simmondsii* [11, 39]. Usually, paralogous *CYP* genes provide functional redundancy during both biotrophic and necrotrophic stages of infection [14], and, although some paralogous *CYP* genes display an apparent subfuctionalization, there are no experimental data available that support this asseveration assertion yet.

Currently, the exhaustive exploration of the function of the CYPs of *Colletotrichum* has not been unexplored. However, the genomic resources that are now available for many *Colletotrichum* species provide an opportunity to allow the formulation of new hypotheses focused on explaining the biological significance of the Cytochrome P450 complement CYPome in the genus *Colletotrichum*. For this work, we used as model the genomic and proteomic data from *C. graminicola*, a fungal maize pathogen that provokes severe losses in the Americas and Europe [3, 40, 41] as well as other species belonging to Graminicola s. c. [13]. Based on comparative genomics, phylogenetic, and transcriptomic analyses, we performed an exploration of the evolutionary scenarios involved in the evolution of CYPs in the Graminicola species complex (s. c.), with the aim of inferring the putative functional roles of these protein families during the fungal infection into the plant, as well as the biological significance of the wide expansion of these enzyme families.

## Results

### Distribution and frequency of the CYPs in *Colletotrichum* genome projects

**Fig. 1:**
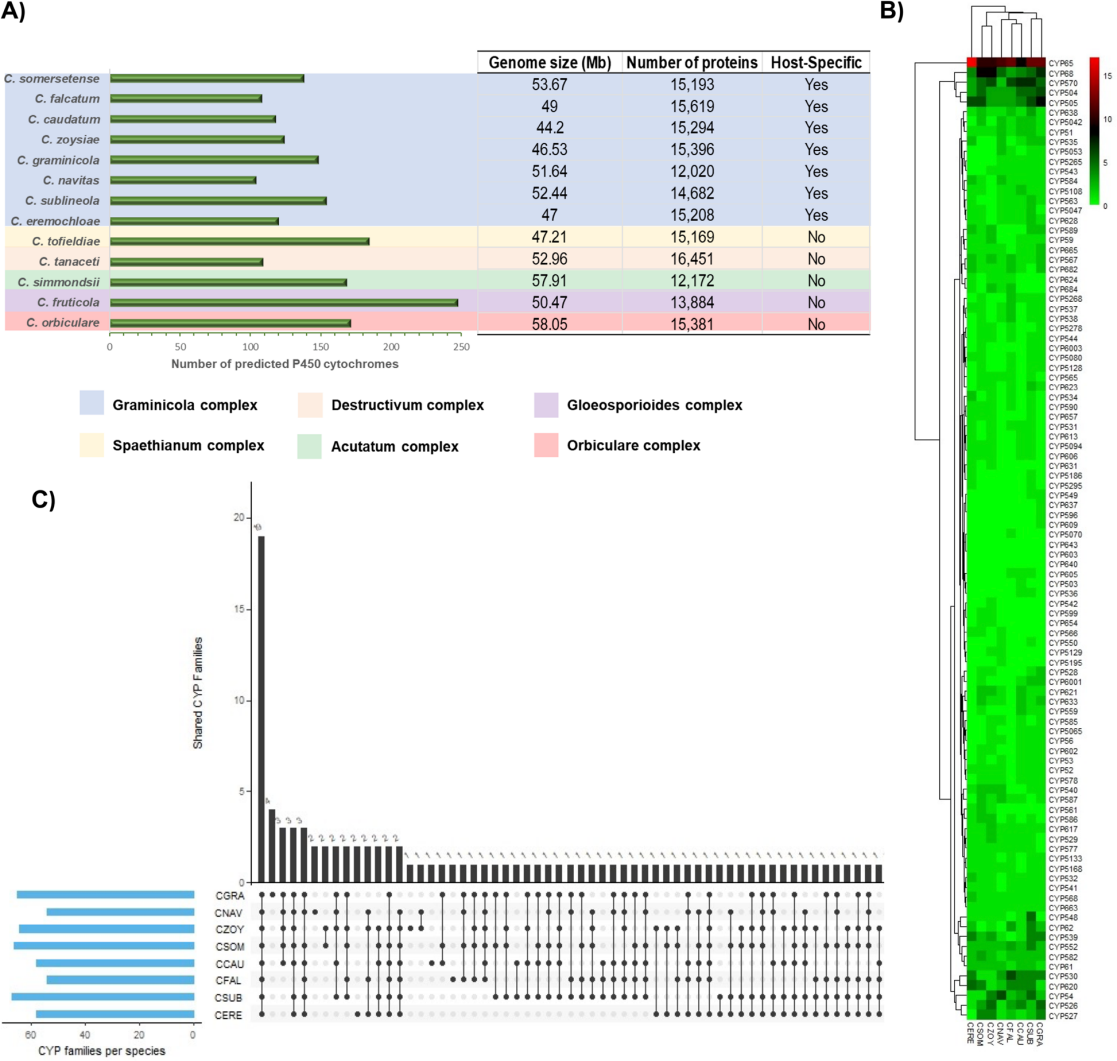
Comparative analysis of the CYPome harbored in the *Colletotrichum* spp. genomes. **A** Comparison of the genome size, protein containing and their correlation with their host-specificity. Background colors highlight the representative species belonging to each complex. **B** Hierarchical clustering comparison of the p450 enzymes that conform the CYPome of each member belonging to Graminicola s. c. The number and classification of each CYP family was visualized employing a heatmap created with the “pheatmap” package included in R program. The color scale in the bar (black, green, and red) represents the protein abundance in each CYP family. **C** Determination of the number of each CYP family shared in each fungal species from the Graminicola s.c. The figure was created by using the R program. Black bars and black points represent the total number of CYP families shared. The blue bars represent the total CYPs contained in each organism. Abbreviations: CCAU, *C. caudatum*; CERE, *C. eremochloae*; CFAL, *C. falcatum*; CGRA, *C. graminicola*; CNAV, *C. navitas*; CSOM, *C. somersetense*; CSUB, *C. sublineola*; CZOY, *C. zoysiae*; CORB, *C. orbiculare*; CFRU, *C. fructicola*; CTOF, *C. tofieldiae*; CTAN, *C. tanaceti*; CSIM, *C. simmondsii*

A total of 150–250 amino acid sequences were identified as members belonging to P450 superfamily in each of the *Colletotrichum* genomes (Fig. 1a; Table S1). From all species belonging to the Graminicola s.c., *C. sublineola*, *C. graminicola*, and *Colletotrichum somersetense* possess the greatest variety of CYPs, however, unlike other species, the Graminicola s. c. exhibited a low content of CYPs (< 150 copies) (Fig. 1a; Table S1). In contrast, *Colletotrichum orbiculare*, *Colletotrichum tofieldiae* and *Colletotrichum fructicola* genomes encode 170, 190 and 250 P450 copies respectively, perhaps associated with adaptation to several plant-physiological conditions (Fig. 1a; Table S1). A total of 98 CYP families were identified from the eight members from the Graminicola s. c. tested. Among them, 63 CYP families were singletons, which were uniquely found in one of the eight species without any paralogs. In addition, 21 families harbored only two copies, 10 families possess among 3–5 copies, and four CYP families correspond to multicopy gene families with more than 5 copies into the eight species genomes (Fig. 1b; Table S2). The CYP65, CYP68, CYP526 and CYP570 families exhibited the largest content of paralog copies, harboring between 5–18 copies (Fig. 1b; Table S2). Likewise, *C. graminicola*, *C. somersetense* and *C. zoysiae*, exhibited the most diversity of CYP families (Fig. 1b). In contrast, a reduced content of CYP families were observed in *Colletotrichum falcatum* and *Colletotrichum navitas*. The comparative analysis revealed that 18 CYP families are shared among the Graminicola s. c. The CYP families corresponding to CYP52, CYP54, CYP507, CYP530, CYP531, CYP58, CYP559, CYP65 contain the largest number of paralogs in the analyzed genomes. Nonetheless, the CYPome of the Graminicola s. c. was shaped by clans containing non-duplicated CYP families, meanwhile only a few families contain large numbers of paralogs (Fig. 1b; Table S2). Most of the expanded CYP families (> 3 copies), such as CYP65, CYP68, CYP526 and CYP570 were present in all fungal species evaluated (Fig. 1c; Table S3). Some CYP families were found to be restricted to the Graminicola s. c.; for instance, *C. graminicola* harbored 3 species-specific CYP families, while the rest of the members harbored one species-specific CYP family (Fig. 1c; Table S3). The classification of the CYPs into functional clans, according to the pipeline described by the Fungal Cytochrome P450 Database (FCDB) revealed that most of them were predicted to be involved in xenobiotic and secondary metabolism (Table 1).

**Table 1:** SMB clusters detected in the genome project of *C. graminicola* strain M1.001. The analysis was performed by using antiSMASH software and carrying out a deep exploration of the genomic resources harbored in NCBI and Mycocosm database

**CYP Clan**	**CYP Family**	**Putative functions**
CYP54	CYP503, CYP54, CYP599, CYP602	Secondary metabolism
CYP504	CYP504	Xenobiotic metabolism
CYP5042	CYP5042	Non determined
CYP505	CYP505, CYP541	Xenobiotic metabolism
CYP507	CYP527, CYP535, CYP570	Xenobiotic metabolism
CYP530	CYP5065, CYP530, CYP663, CYP665	Xenobiotic metabolism
CYP531	CYP5080, CYP631	Xenobiotic metabolism
CYP546	CYP5053	Non determined
CYP547	CYP5070, CYP582, CYP617	Secondary metabolism
CYP58	CYP5094, CYP542, CYP552, CYP682	Secondary metabolism
CYP51	CYP51	Primary metabolism
CYP5108	CYP5108	Non determined
CYP52	CYP52, CYP538, CYP539, CYP584, CYP585	Xenobiotic metabolism
CYP526	CYP526, CYP638	Secondary metabolism
CYP528	CYP528	Non determined
CYP53	CYP53	Xenobiotic metabolism
CYP531	CYP531, CYP532, CYP536	Xenobiotic metabolism
CYP534	CYP534	Non determined
CYP537	CYP537, CYP577	Xenobiotic metabolism
CYP540	CYP540	Primary metabolism
CYP529	CYP543	Non determined
CYP544	CYP544	Non determined
CYP548	CYP548	Xenobiotic metabolism
CYP549	CYP549	Non determined
CYP550	CYP550, CYP633	Secondary metabolism
CYP559	CYP559, CYP606, CYP623	Non determined
CYP56	CYP56	Primary metabolism
CYP65	CYP561, CYP563, CYP565, CYP567, CYP568, CYP65	Secondary metabolism
CYP566	CYP566	Non determined
CYP578	CYP578	Secondary metabolism
CYP59	CYP586, CYP587, CYP59	Secondary metabolism
CYP589	CYP589	Non determined
CYP590	CYP590	Non determined
CYP68	CYP596, CYP68	Secondary metabolism
CYP605	CYP605	Secondary metabolism
CYP609	CYP609	Non determined
CYP61	CYP61	Primary metabolism
CYP613	CYP613	Secondary metabolism
CYP62	CYP62, CYP684	Non determined
CYP533	CYP620, CYP621	Xenobiotic metabolism
CYP624	CYP624	Non determined
CYP574	CYP628	Secondary metabolism
CYP637	CYP637	Non determined
CYP653	CYP653	Secondary metabolism
CYP657	CYP657	Primary metabolism

CYP’s did not have match with any clan harbored in FCPD: CYP5047, CYP5128, CYP5129, CYP5133, CYP5168, CYP5183, CYP5195, CYP5278, CYP5295, CYP6001, CYP6003

### Phylogenetic relationships between paralogous CYP families

**Fig. 2:**
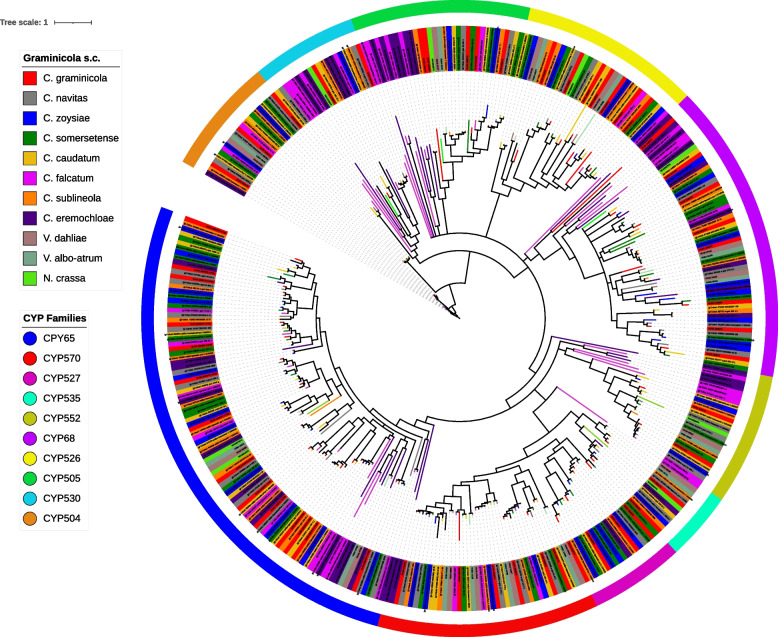
Bayesian phylogenetic tree constructed with amino acid sequences from representative multi-extended paralogous CYP families. The LG + I + G evolutionary model was selected for the phylogenetic reconstruction. The scale bar represents the number of substitutions per site

The phylogenetic divergence in paralogous CYP’s was analyzed employing the 10 highly expanded CYP families. We analyzed the evolutionary changes and origin of the paralogous members of CYPs using as models the families CYP504, CYP505, CYP526, CYP527, CYP530, CYP535, CYP552, CYP570, CYP65, and CYP68, because the biological functions of these families have been well described and characterized in other plant pathogenic fungal species [25, 28]. The phylogeny displayed numerous orthologous groups clustered in the phylogeny (Fig. 2). In general, the CYP families were gathered as orthologous groups that possess a consistent with the phylogenetic relationships among members of the Graminicola s. c. Also, it was observed that CYP65, CYP68, CYP505, CYP552 belonging to *C. falcatum* and *C. eremochloae* presented additional duplication events with a putative recent appearance, hence they were cataloged as orthologous/paralogous groups, although this putative duplication event is not in all the phylogenetic tree (Fig. 2). In fact, de distribution of the several orthologous groups belonging to a same families could be associated to an ancient paralogous duplication event (Fig. 2). On the other hand, paralogs from internal duplications were not observed in the rest of Graminicola s.c (Fig. 2). Most of the CYP displayed short branch lengths compared with their clustered orthologs (Fig. 2). Also, the phylogenetic tree showed that CYP65, CYP527, CYP570, CYP535 and CYP552 families are closely related, forming a unique evolutionary clade. On the other hand, CYP504 was recognized as the oldest family in the phylogeny, due to its localization near the root (Fig. 2). The CYPome belonging to the closely related fungi used as external group (*Neurospora crassa*, *Verticillium dahliae* and *Verticillium albo-atrum*) exhibited orthologs paralogous CYP family tested in this analysis. Nevertheless, these species displayed few paralogous CYP’s: *N. crassa* clustered two CYP52; *V. dahliae* clustered two CYP526, two CYP68, three CYP570 and two CYP65; *V. albo-atrum* clustered two CYP526 and four CYP570 (Fig. 2).

### Expansion and contraction patterns of CYP families among Graminicola s.c.

**Fig. 3:**
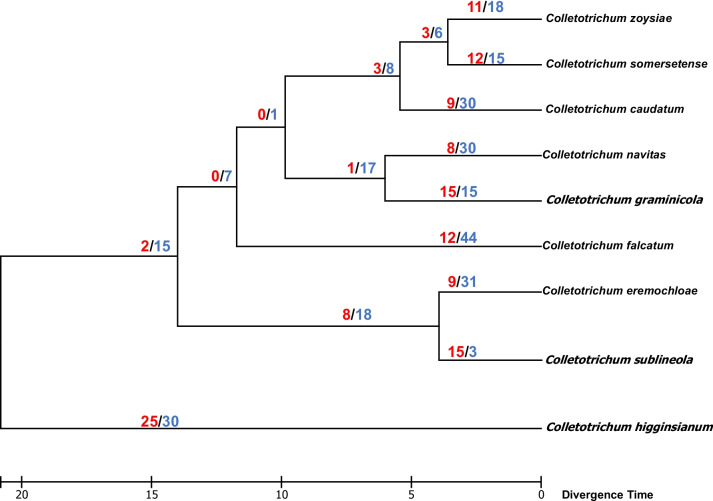
Gene gain and loss patterns in species belonging to *Colletotrichum* Graminicola s. c. Phylogenomic tree was constructed based on distance-methods by using 1765 single-copy orthologous genes. Divergence times were estimated with MEGAX. Numbers at the nodes represent expansion (red) and contraction (blue) events

An analysis of gene family evolution was carried out using the software CAFE. In general, the analysis revealed that the Graminicola s. c. clade exhibits a greater number of family contractions than the number of family expansions, except for *C. sublineola* CBS 129661 where the number of expansions were predominant (Fig. 3; Table S4). In fact, all species except *C. sublineola* CBS 129661 displayed more families experiencing contractions than the family’s undergoing expansion. In *C. graminicola*, the number of expansions and contractions was maintained in the same proportion, unlike that of *C. navitas*, where the gene loss is indeed more dominant than duplications. Other species that kept a similar proportion of expansions/contractions were *C. somersetense* and *C. zoysiae*. Regarding to the *CYP* gain and loss, we observed that the family expansion occurred families CYP5070, CYP635, CYP637, and CYP654 (Table S6). The branches that contain the common ancestors of the Graminicola s. c. clade also presented more predominance of gene family contractions and no expansions in some nodes particularly in the branch belonging to the ancestor of *C. graminicola* and *C. navitas*, and the branch corresponding to the common ancestor of *C. eremochloae* and *C. sublineola* strains, the proportion of expansions/contractions indicate a greater amount of gene losses than gene gains (Fig. 3; Table S4).

### Diversity and conservation of the motif sites

**Fig. 4:**
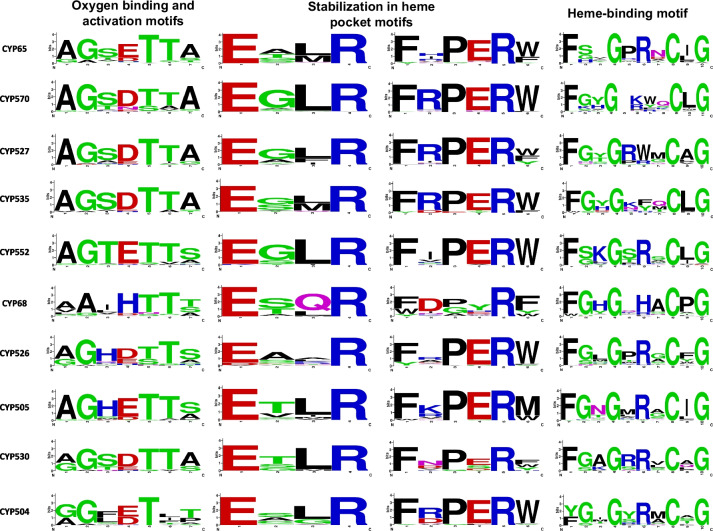
Sequence logos from conserved motive sites harbored in representative paralogous CYP families. Alignments were performed with MUSCLE v3.8.3 software. The consensus logos images were generated by using WebLogo webserver

In general, the sequence logos revealed moderate conservation of the motif sites (Fig. 4). The motif AGXTTXX associated with oxygen binding displayed ~30% of amino acid variations in their most conserved sequence sites located at positions 1, 2 and 6. Approximately, 50% of amino acid variations in this motif were observed at position 3, 4 and 7. Position 5 in motif AGXTTXX was the most conserved motif displaying only a replacement in CYP68 and CYP570 (Fig. 4). The EXXR motif responsible for the stabilization of heme pocket site exhibited conservative amino acids at position 1 and 4, while the positions 2 and 3 displayed also conservative variabilities, with exception of position 3 in CYP68 family, which presented evidence of amino acids with non-shared chemical structure properties L and Q [42]. Likewise, most of the positions at PER sites were maintained well conserved, except for position 2, which displayed high variability in all families. Particularly, CYP68 showed non-conservation in the PER site (Fig. 4). In general, the heme-binding site (FXXGXRXCXG) displayed conservation at position 1, 4, 8 and 10, however, the rest of the positions have high variability (Fig. 4).

### Expression of the CYPome during the infection stages in *C. graminicola* M1.001

**Fig. 5:**
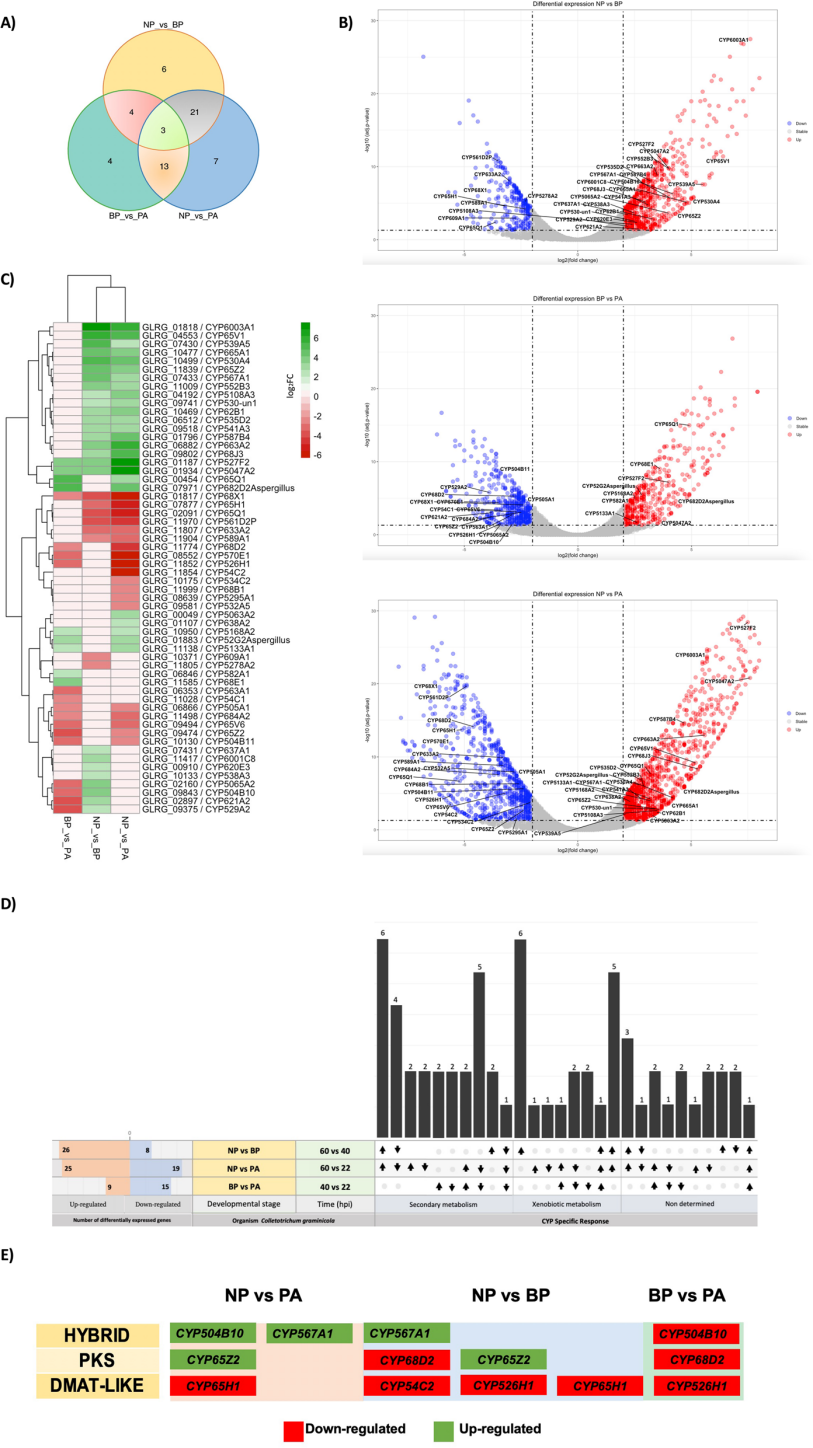
Transcriptome analysis for differentially expressed genes in three stage developmental stages of infection in *C. graminicola* M1.001 associated with CYPome. **A** Venn diagram shows common and unique genes for all three comparisons. **B** Volcano Plots show *p-value* and Fold Change for each pairwise comparison with ID CYP for each differential expressed gene, where red is up-regulated, and blue is down-regulated. **C** Heatmap with cluster showing the pattern of expression in the three pairwise comparisons, CYP genes are clustered, and the color gradient is the Fold Change value, where green is up-regulated, and red is down-regulated. **D** UpSetR displays are grouped differential expressed genes for all pairwise comparisons with an intersection about the metabolism (Secondary metabolism, Xenobiotic metabolism, and non-determined metabolism). NP = 60 h post-infection (hpi), in necrotrophic phase. BP = 40hpi, in biotrophic phase. PA = 22 hpi, in appressorium phase

To identify differentially expressed *CYP* genes during the main infection stages of infection, we reanalyzed the previously published RNA-seq data by focusing only on the genes associated to CYPome [14]. We determined a subset of 58 Differentially Expressed Genes (DEGs) that are the common among the three proposed pairwise comparisons, these three results follow the same comparisons of the O’Connell’s analysis (Fig. 5a and b). As shown in Fig. 5a, 3 DEGs were detected in common in the three pairwise comparison GLRG_01187 (CYP527F2); GLRG_01934 (CYP5047A2); GLRG_01817 (CYP68X1). Two of these are up-regulated while the GLRG_01817 gene is repressed (Fig. 5c). Interestingly, the genes GLRG_02160 (CYP5065A2), GLRG_09843 (CYP504B10), GLRG_02897 (CYP621A2), GLRG_09375 (CYP529A2) were suppressed in biotrophic phase and the same genes were up-regulated in the necrotrophic phase, but these 4 genes were not differentially expressed when compared in the appressorium and necrotrophic phase (Fig. 5c). Currently, research on the virulence role of the P450 genes is poorly studied, but these results suggest that these 4 genes may be involved in virulence, playing a key role in the necrotrophic phase of this hemibiotrophic fungus. A total of 26 up-regulated DEGs and 8 DEGs down-regulated were detected at the Necrotrophic Phase (NP) vs Biotrophic Phase (BP) comparison, from a total of 13 DEGs in relation to secondary metabolism, 12 DEGs in xenobiotic metabolism, and 9 expressed genes which were annotated as non-determined for a *CYP* specific response (Fig. 5d). In the case of NP vs Appressorium phase (PA) comparison, 44 DEGs were detected, as well as 19 down-regulated and 25 up-regulated, which 22 *CYP* genes were related with secondary metabolism, and 11 DEGs with xenobiotic metabolism, but 11 *CYP* genes were classified as non-determined for any category. In the compassion of BP vs AP 24 DEGs were found, 15 down-regulated and 9 up-regulated, of which, 12 were related with secondary metabolism, and 6 DEGs with xenobiotic metabolism, 6 with no associated metabolism category. (Fig. 5d). The genes related to secondary metabolism were more predominant with a total of 28 genes, within this category. Some DEG’s *CYPs* were recognized as members of Polyketide synthase (PKS) and PKS-Non-Ribosomal Peptide Synthetase (NRPS) (HYBRID) clusters (Fig. 5e). Two *CYP*s located into the HYBRID clusters and one *CYP* located in a PKS cluster displayed up-regulation at the NP vs PA, while two *CYPs* cataloged as members of HYBRID and PKS cluster respectively during the NP vs BP were up-regulated, and all *CYP*s classified into Dimethylallyl tryptophan synthase -like cluster (DMAT) were defined as down-regulated genes in all conditions. On the other hand, all *CYPs* harbored in Secondary Metabolite Gene Clusters (SMGCs) displayed down-regulation when BP vs AP conditions were evaluated. This first summary of RNA-Seq data [14] in three stages of disease development showed the involvement of only *CYP* genes during pathogenicity of *C. graminicola* M1.001 thus explaining the role of *CYP* families in plant-pathogen interaction.

### Structural evolution of the paralogs CYP enzymes of *C. graminicola* M1.001

**Fig. 6:**
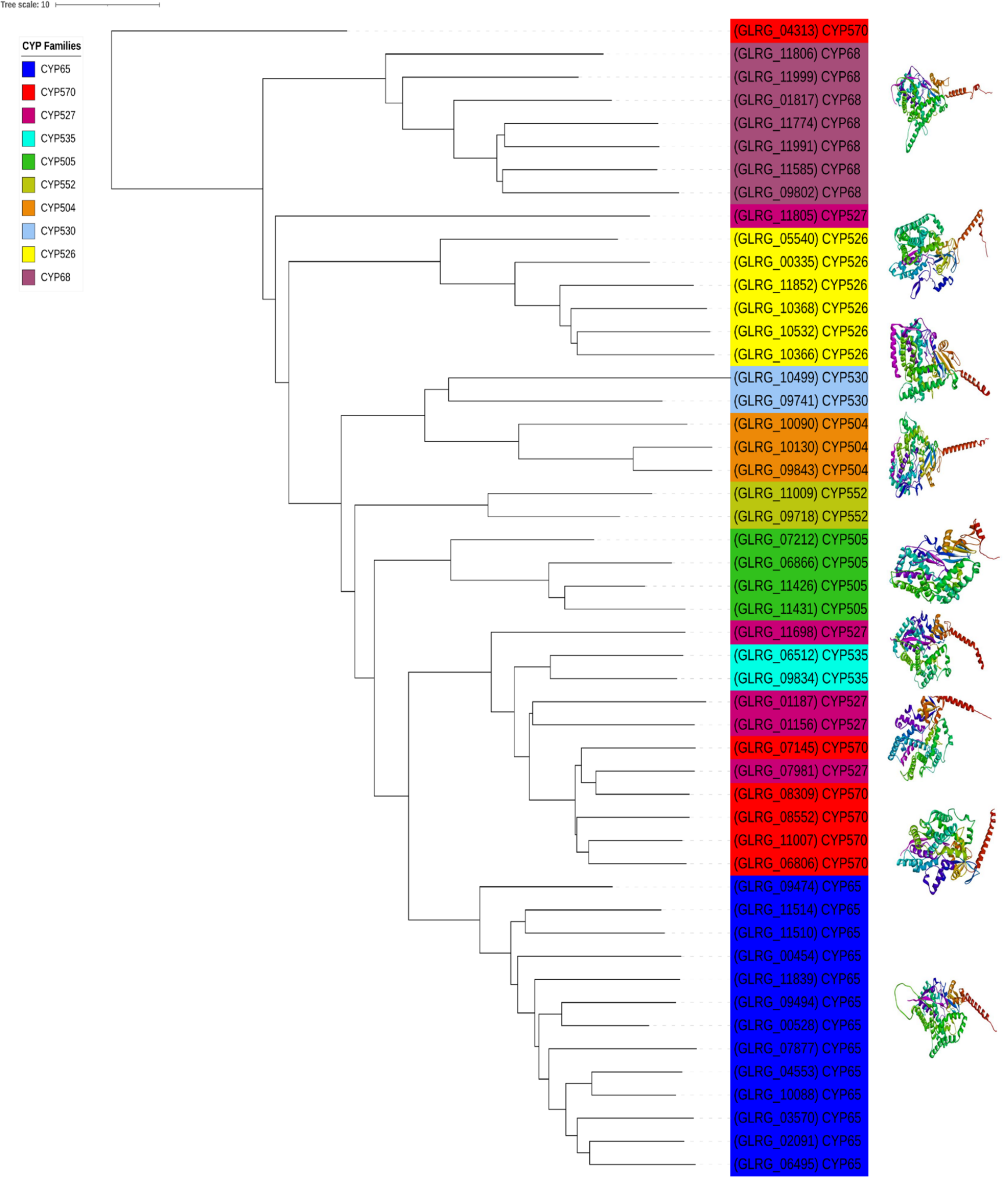
Phylogeny constructed with the hypothetical three-dimensional (3D) structures of representative paralogous CYP families in *C. graminicola* M1.001. The phylogenetic tree represents the evolutionary relationships among CYP families based on 3D-structural similarities. The phylogeny was constructed employing hypothetical three-dimensional structures generated with Alphafold2 software and computed with DALI software

We performed an analysis to evaluate the hypothetical structural evolution using a 3D-structure-based phylogeny. The evolutionary structural relationship among paralogous CYP enzymes belonging to *C. graminicola* is shown in Fig. 6. In the phylogeny, almost all paralogous CYP families clustered in a highly similarity topology compared with the amino-acid sequence-based phylogeny (Figs. 2 and 6). Nevertheless, some paralogs belonging CYP527 and CYP570 clustered with other paralogs displaying a putative structural evolutionary convergence. Thereby, most of the paralogous CYP members show a common folding origin, but CYP527 and CYP570 experienced polyphyletic structural evolution (Fig. 6). Additionally, the Structural-Based Dendrogram (SBD) topology reflects that of the CYP families, and there are some paralogous enzymes of the same family that exhibit differences in the structural domain architectures (Fig. 6).

### Diversity of CYP families harbored in SMGCs and syntenic conservation

**Fig. 7:**
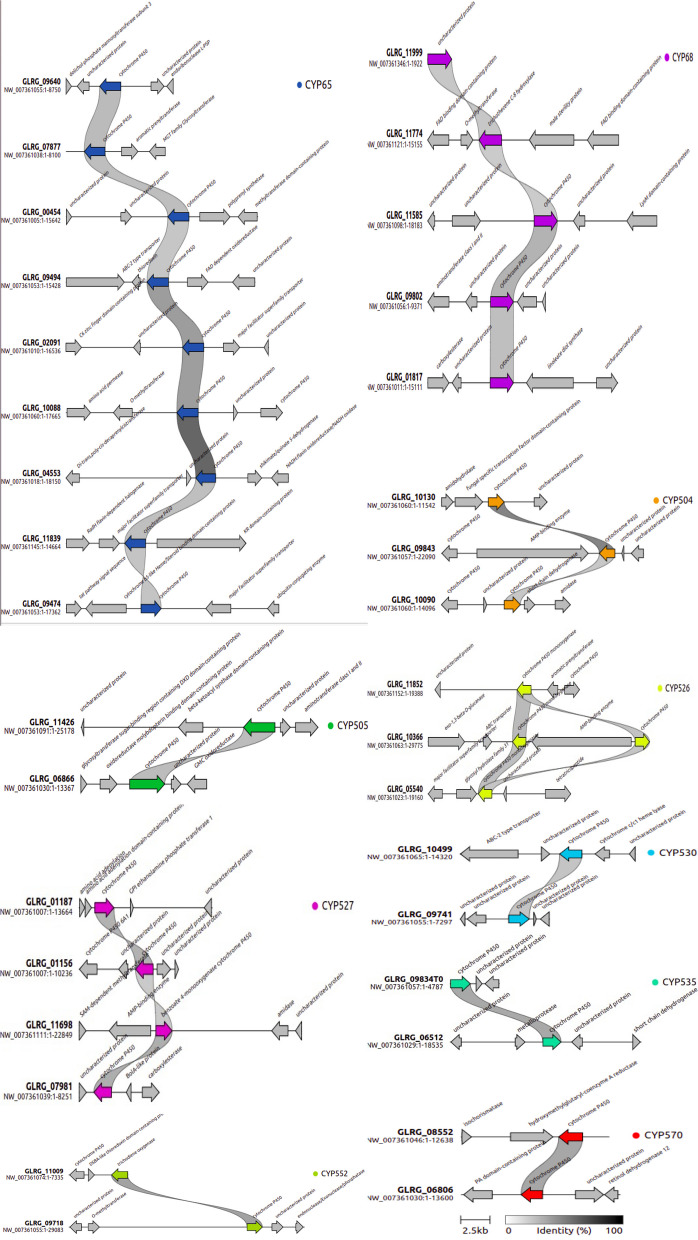
Synteny conservation of CYP genes harbored in Secondary Metabolite Gene Clusters (SMGC) in *C. graminicola* M1.001. Legends at the bottom represent the functional assignation of the genetic elements found in the clusters. Synteny analysis was performed by using the MAUVE program included in the Geneious Prime software

A total of 30 *CYP* genes were identified inside the SMGCs recognized in the genome project of *C. graminicola* M1.001 (Table 2). Most of the *CYP* genes were recognized as paralogous members belonging to *CYP65, CYP68*, *CYP526*, *CYP527*, *CYP539*, *CYP570* and *CYP682* families. Four *CYP65* genes were detected into SMGCs clusters (Table 2). PKS clusters exhibited that harbor 11 *CYP* genes, while one and two *CYPs* were present in the siderophore and NRPS-like clusters respectively (Table 2). On the other hand, the number of CYPs in DMAT and HYBRID clusters correspond to three and five genes respectively (Table 2). All paralogous *CYPs* of *C. graminicola* M1.001 were harbored in SMGCs that display an evident loss of synteny. Besides, the genomic arrangements of the clusters indicate that several *CYP* paralogous members are in regions alongside genes not associated with SMGCs (Fig. 7).

**Table 2:** Hypothetical functional assignation of CYP families. The assignment was performed based on the analysis described by [25]

**Gene ID**	**CYP gene**	**SM cluster**	**CLUSTER ID**	**Source**
GLRG_09843T0	CYP504B10	HYBRID	Colgr1.5	Mycocosm/AntiSMASH
GLRG_10366T0	CYP526H1	HYBRID	Colgr1.7	Mycocosm/AntiSMASH
CLGR_10368T0	CYP526A1	HYBRID	Colgr1.7	Mycocosm/AntiSMASH
CLGR_10371T0	CYP609A1	HYBRID	Colgr1.7	Mycocosm/AntiSMASH
GLRG_07433T0	CYP567A1	HYBRID	Colgr.1.46	Mycocosm/AntiSMASH
GLRG_11698T0	CYP527F1	NRPS-like	Colgr1.52	Mycocosm/AntiSMASH
GLRG_01187	CYP527F2	NRPS-like	NW_007361007	NCBI
GLRG_08552	CYP570E1	Siderophore	NW_007361046	NCBI
GLRG_11852T0	CYP526H1	DMAT-like	NW_007361152	NCBI
GLRG_11854T0	CYP54C2	DMAT-like	NW_007361152	NCBI
GLRG_07877T0	CYP65H1	DMAT-like	Colgr.45	Mycocosm/AntiSMASH
GLRG_10319T0	CYP585A3P	PKS	Colgr1.6	Mycocosm/AntiSMASH
GLRG_11880T0	CYP589A1	PKS	Colgr1.9	Mycocosm/AntiSMASH
GLRG_11881T0	CYP539C1	PKS	Colgr1.9	Mycocosm/AntiSMASH
GLRG_10534T0	CYP682B3	PKS	Colgr1.10	Mycocosm/AntiSMASH
GLRG_10536T0	CYP596B1	PKS	Colgr1.10	Mycocosm/AntiSMASH
GLRG_11562T0	CYP606B2	PKS	Colgr1.19	Mycocosm/AntiSMASH
GLRG_09074T0	CYP68B1	PKS	Colgr1.21	Mycocosm/AntiSMASH
GLRG_03513T0	CYP665A1	PKS	Colgr1.26	Mycocosm/AntiSMASH
GLRG_11834T0	CYP539C1	PKS	Colgr1.30	Mycocosm/AntiSMASH
GLRG_11839T0	CYP65Z2	PKS	Colgr1.31	Mycocosm/AntiSMASH
GLRG_11774T0	CYP68D2	PKS	Colgr1.40	Mycocosm/AntiSMASH
GLRG_10532T0	CYP526A1	PKS-like	NW_007361066	NCBI
GLRG_10534T0	CYP682B3	PKS-like	NW_007361066	NCBI
GLRG_07145T0	CYP570C4	PKS-like	NW_007361033	NCBI
GLRG_08309	CYP570B1	PKS-like	NW_0077361042	NCBI
GLRG_11426	CYP505C1	PKS-like	NW_007361091	NCBI
GLRG_09718	CYP552B1	PKS-like	NW_007361055	NCBI
GLRG_00454	CYP65Q1	PKS-like	NW_007361005	NCBI
GLRG_11514	CYP65AK2	PKS-like	NW_007361094	NCBI

## Discussion

Although *Colletotrichum* species exhibit a large CYPome shaped by many families [38], assigning the specific biological roles to each member is a research challenge. According to our analysis, some members from *Colletotrichum* genus have larger CYPome in terms of the number of CYP elements than other plant pathogenic fungi such as *Cryphonectria parasitica* (70 CYP members), *M. oryzae* (77 CYP members) or *Aspergillus* sp. (57–92 CYP members), whose CYPomes are well documented in the fungal CYP database (http://p450.riceblast.snu.ac.kr/). However, in terms of gene gain and loss, Graminicola s. c. experienced a reductive evolution in several families of its CYPome, but the presence of a large number of CYPs is derived from an extensive appearance of paralogs in certain CYP families, and the maintenance of several CYP families. This information suggests that the presence of a broad CYPome possibly plays a critical role upon the optimization of the colonization capability and virulence in plant pathogenic fungi, resulting in its significant importance for the adaptation of pathogens during plant invasion [43, 44].

The size of CYPome from Graminicola s. c. is reduced in comparison with the number of CYP enzymes identified in *Colletotrichum* species from Spaethianum, Acutatum, Gloeosporioides and Orbiculare s. c., despite displaying a proteome with a similar or even larger size than the proteomes from the species belonging to the complexes described above. Hence, it is suggested that CYPome of the members of the Graminicola s. c. were subjected to gene losses during their evolutionary history. In addition, the members of this complex suffered several gene losses in other families associated with virulence such as lineage specific candidate effectors and CAZY enzymes, indicating that the contraction of the CYPome may be associated mainly to host range and specificity [11, 13, 39]. Although there is non-specific gene family expansion associated with host range, regularly eudicot infecting species have a higher overall number of gene families than monocot infecting species [13]. According to our results, eudicot-infecting *Colletotrichum* species*,* for instance *C. fructicola* possess very expanded CYPomes in terms of number of CYP. Therefore, our results are consistent with the hypothesis monocot pathogens have lower diversity of CYPome encoding genes than eudicot infecting *Colletotrichum* species [13]. Moreover, these fungi present a broad expansion of their virulence related gene families [39]. Earlier studies also affirmed that protein gains and losses are associated with their host range and specificity [11, 45].

Unfortunately, most of these families lack experimental evidence to confirm their functions. Nevertheless, according to the CYP classification [25, 46], we predicted functions based on similarity and functional domain content (Table S5), where most of the CYPome of members belonging to the Graminicola s.c. might be involved in the synthesis of secondary metabolites, since, based on previous experimental evidence, several families present in the genomes of these fungi participate in xenobiotic metabolism [47]. In other fungi, for instance *Fusarium* and *Trichoderma* spp., several members of the CYPome are associated in cell detoxification and production of fungal compounds [28, 48]. We propose that the putative function of the CYPome of the Graminicola s. c. members is associated with the biosynthesis of toxins or chemical modification of compounds secreted by the host.

In *Trichoderma* spp. the expanded CYPome enabled their survival in their respective habitats [48]. The genus *Fusarium* is shaped by several species of plant pathogens that also exhibit a saprophytic stage. The presence of a wide CYPome in *Fusarium* spp. is not only associated with virulence, since there are CYPs whose function is closely related with sexual spore development [49]. In the case of *Colletotrichum* spp., its CYPome may exhibit functional roles resembles to of *Trichoderma* spp. and *Fusarium* spp., thereby the evolution of CYPome of *Colletotrichum* might be influenced by the selection pressures exercised during their adaptation as pathogens. The expansion of CYPome may allow them to be more efficient during their invasion of the host.

The CYPome of members of the Graminicola s. c. are composed mostly of single-copy families. The multi-copy families, even though they represent a low percentage of the total CYPome, are very expanded by most species. In fact, the most expanded CYPs families are conserved into all Graminicola s. c. Most of the CYPome display families that cluster only unique copies or duplicated genes, nonetheless these families were not present in all the analyzed species. In fact, various single-copies CYP families may be catalogued as species-restricted families. Species-restricted CYP families have been reported in Basidiomycete biotrophic plant pathogens, where these genes may fulfill an important role in the host invasion [50]. Also, the presence of species-restricted families is very common in pluricellular organisms, for instance as water strides, wherein these gene families have allowed several evolutionary advantages such as a better locomotion [51]. The presence of species-restricted genes confers adaptive advantages in the organisms for phenotypic novelty [52, 53]. Our findings suggest that, although the maintenance of multi-copy families indicate that they are critically essential for Graminicola s. c members, the presence of species-restricted families suggest not only specific gene loss events but also that the retained CYPs are especially important for their lifestyle. The species restricted CYPs play new functions in the *Colletotrichum* Graminicola s.c.

The paralogous CYP families employed for this study possess the greatest number of orthologous and paralogous members; thus, it is important to inquire about the most accurate biological background that prompted their expansions. CYP504 family has important roles in xenobiotic metabolism in *Trichoderma* species, as well as CYP504 enzymes in *Aspergillus nidulans* participates in the degradation of phenylacetate as sole carbon source [48, 54]. CYP505 family are membrane-associated cytochromes capable of hydroxylate fatty acid [55]. The expression of CYP505 allows plant-pathogen fungi, for instance *Fusarium oxysporum* to employ oxidized fatty acids for inactivating plant defense system [56]. The members CYP526, CYP65 and CYP68 families underlie the biosynthetic pathway of trichothecene mycotoxins in *Fusarium* sp. [28, 57]. The CYP527, CYP530, CYP535 and CYP570 families have displayed putative roles in xenobiotics metabolism [25, 48]. The presence of members belonging to the CYP552 family confers fungal protection against toxic compounds secreted by the host, however, CYP552 cytochromes also may underlie secondary metabolites pathways [25, 58].

All paralogous CYPs families described above are highly expanded and conserved among Graminicola s. c members, which means that they already were present in the common ancestral *Colletotrichum* species, and their expansion may be a consequence of multiple ancestral and modern duplications that occurred both in the common ancestor and after the speciation event. However, there is a small number of species-restricted paralogous detected in the CYPs of study. Hence, the events of duplications in multi-protein CYPs were complemented by a set of gain and loss protein processes. This evolutionary scenario has already been well documented in some P450 cytochromes, such as members of CYP52 family in Saccharomycetales yeast [59]. In fact, the CYPs contraction was more pronounced in some members of the Graminicola s. c. The gene loss events occurred in several *Colletotrichum* species already described in earlier studies have mentioned the loss of orthologous and paralogous members into the multigene CYPs [28, 45]. On the other hand, the phylogenetic distribution suggests that CYP504 evolved early, while other families such as CYP65, CYP570, CYP527, CYP535 and CYP552 are closely related, and their origin is more recent.

Remarkably, in *C. graminicola*, some members belonging to distinct paralogous CYP families display different phylogenetic relationships at tertiary structure level, since those paralogous CYP’s display structural similarities among them, indicating a putative event of convergent/parallel evolution. This evolutionary event detected in CYPs belonging to *Colletotrichum* is not an unusual phenomenon, rather it is quite common among the P450 superfamily members [16, 60]. For instance, CYP384 and CYP2J19, two enzymes from *Tetranychus kanzawai* and canaries respectively, do not share a common lineage, however both families have similar folding and thus enzymatic activity [61]. This example of convergent evolution enabled the early diverging CYPs to have similar functions. This phenomenon has been identified in P450s of the CYP52 family, which have affinity by a wide range of alkanes and fatty acids but maintain some functional moieties [59]. Is important to emphasize that.

The presence of a high number of paralogous proteins harbored into CYPs is associated mainly with evolutionary process for adapting to niches frequently exposed to several environmental and nutritional conditions [59]. It is still unclear why certain CYPs in *Colletotrichum* and other fungal species are highly expanded. In mammals, the expansion of the opsin gene family, which encodes a protein that is located in photoreceptor cells of primates, allows them to distinguish a wide spectrum of wavelengths [62, 63]. Hence, the high fate of duplication in many CYP families may contribute as an adaptation mechanism for increasing or improvement of their virulence. Nonetheless, it is of utmost importance to inquire which is the biological role of each paralogous. Exploring the transcriptional profiles of the CYPome based on raw data obtained previously [14] by using as model *C. graminicola* M1.001, 59 *CYP* genes belong to *C. graminicola* showed upregulation and downregulation profiles in the different stages of infections. In fact, most of this cluster of *CYP* genes were overexpressed during necrotrophic phase, while various *CYPs* were repressed in the biotrophic phase. These findings suggest that *CYPs* may play a fundamental role in the cellular and tissue destruction of the plant host by *C. graminicola*. However, several paralogous, members of CYP65, CYP68, CYP535 and CYP527 display similar expression levels, suggesting that many paralogous are still functionally redundant, although signals of subfunctionalization also might be associated, however, is necessary to obtain more experimental data for confirming this asseveration. Perhaps, the robustness event, a phenomenon that explain the maintaining of redundant paralogous genes in an organism [64, 65], may be responsible of the putative redundancy of orthologous and paralogous members of the CYPome with the purpose of maintaining the normal functions of the fungal cells in the presence of several perturbations during the stages of their lifestyle.

In this work we also examined the genomic context and changes in the motif sequences of the CYPome by using as model multi-protein CYP families. In general, in each CYPs there is a low conservation of the signature features of the motif sites. In particular, the motif sequence FXXGXRXCXG is highly variable, indicating great variations into the catalytic pocket. Likewise, the variations observed in other motif sequences reflect the CYPome belonging to Graminicola s. c have accumulated several mutations along their evolutionary history, by exposition to several pressure selection conditions. These variations could be the result of the adaptation of each CYP for interacting with several substrates, as it was observed into the CYP52 family [59]. On the other hand, genomic rearrangements in the region of the main paralogous P450 families indicate an evident loss of synteny. A probably mechanisms associated with the random location in the genome of the paralogous *CYPs* could be mediated by transposable elements [63, 66], however, more additional experimental information is necessary for confirming this hypothesis. Although some *CYPs* are in SMGCs, most of them are in regions whose genetic elements do not provide clear information about their biological roles.

## Conclusions

The evolutionary analysis of the CYPome of Graminicola s. c provides new insights into the putative environmental phenomena involved in the expansion and contraction events observed in the CYPome of *Colletotrichum*. Even though this study allowed to inquire that CYPome may be an essential role in the biosynthesis of secondary metabolites, antifungal compounds, or confers protection against plant defense mechanisms during the interaction the host, it is necessary to confirm this through further laboratory experiments. CYPome of Graminicola s.c. suffered reductive evolution, expanding single gene CYP families and conserving few families containing multiple paralogous. In fact, we suggest that CYPome suffered several gene loss events after an extensive duplication event. On the other hand, although the CYPome of Graminicola s.c. suffered some depurative events, some CYP families generated many paralogs to compensate for the possible negative effects of the gene losses. Therefore, the CYPome in our organisms of study is result of an evolutive process for conferring a better fitness in the members of Graminicola s. c.

## Methods

### Genome project data

Proteomes from genome projects of 8 species/strains from the Graminicola s.c. were used for this study [13, 14, 45, 67–69]. One representative genome project belonging to the *Colletotrichum* species complex (s.c.) closely related to Graminicola s.c. was chosen for some comparative analysis (Table 3).

**Table 3:** List of *Colletotrichum* spp. genome resources used in this study

**Abbr.**	**Strain**	**Organisms**	**Species complex**	**Host**	**Origin**	**Accession Nº**	**Database**	**Bioproject**	**Reference**
CCAU	CBS 131602	*C. caudatum*	Graminicola	*Sorghastrum nutans*	USA	JAHLTY000000000.1	NCBI	PRJNA262368	[13]
CERE	CBS 129661	*C. eremochloae*	Graminicola	*Eremochloa ophiuroides*	USA	JAHMUH000000000.1	NCBI	PRJNA262442	[13]
CFAL	MAFF 306170	*C. falcatum*	Graminicola	*Saccharum officinarum*	Japan	JAHLKA000000000.1	NCBI	PRJNA262221	[13]
CGRA	M1.001	*C. graminicola*	Graminicola	*Zea mays*	USA	ACOD00000000.1	NCBI	PRJNA37879	[14]
CNAV	CBS 125086	*C. navitas*	Graminicola	*Panicum virgatum*	USA	JAHLJV000000000.1	NCBI	PRJNA262371	[13]
CSOM	CBS 131599	*C. somersetense*	Graminicola	*Sorghastrum nutans*	USA	JAHMAF000000000.1	NCBI	PRJNA262441	[13]
CSUB	CBS 131301 v1.0	*C. sublineola*	Graminicola	*Sorghum bicolor*	Burkina Fasso	-	JGI	-	[13, 66]
CZOY	MAFF 235873	*C. zoysiae*	Graminicola	*Zoysia tenuifolia*	Japan	JAHMAF000000000.1	NCBI	PRJNA262217	[13]
CORB	MAFF 240422	*C. orbiculare*	Orbiculare	*Cucumis sativus*	Japan	AMCV00000000.1	NCBI	PRJNA171217	[13, 44]
CFRU	Nara gc5	*C. fructicola*	Gloeosporioides	*Fragaria x ananassa*	Japan	ANPB00000000.1	NCBI	PRJNA171218	-
CTOF	CBS 168.49	*C. tofieldiae*	Spaethianum	*Lupinus polyphyllus*	Germany	LFHQ00000000.1	NCBI	PRJNA286723	[67]
CTAN	BRIP5731	*C. tanaceti*	Destructivum	*Tanacetum cinerariifolim*	Australia	PJEX00000000.1	NCBI	PRJNA421029	[68]
CSIM	CBS 122122	*C. simmondsii*	Acutatum	*Carica papaya*	Australia	JFBX00000000.1	NCBI	PRJNA239224	[13]

### Annotation and classification of the CYP proteins

The annotation and classification pipeline of the CYPome was carried out based on a two-step procedure. First, the annotation step was performed by using Hidden Markov Models with HmmerBuild and HmmerSearch programs included in the HMMER v3.3 package [70]. The seed alignment of domain PF00067 deposited in the Pfam protein family database (www.pfam.xfam.org) was used for protein annotation in the selected fungal proteomes, applying an E value = 10^–4^ as a threshold for the selection of the positive hits. The classification procedure was performed with the BLASTP tool comparing the positive hits against the complete CYP sequence list deposited into the Fungal Cytochrome P450 Database (FCDP) [26]. The family assignation of the CYP enzymes was performed based on the highest similarity percentage (at least 40%) exhibited by each hit during the BLAST analysis, according to the classification parameters established as previously referred [71, 72].

### Phylogenetic analysis of representative multiprotein CYP families

Annotated CYP enzymes were aligned with MUSCLE v. 3.8.31, included in the program SeaView version 4.7 [73, 74]. The selection of the best evolutionary model was computed with ProtTest 3 [75]. A Bayesian MCMC-based phylogenetic tree was constructed with MrBayes version 3.2.7a [76], by using LG + I + G evolutionary model. The BMCMC was run for 3 × 10^6^ generations and sampling every 100 generations. The posterior probabilities threshold was over 75%. The phylogenetic tree was edited with the web server iTOL v3 [77].

### Gene family evolution

CAFE software version 4.2.1 was employed for the gene family expansions/contractions analysis [78]. As part of the analysis, the deduced proteomes of *Graminicola s.c.* were classified into orthogroups with OrthoFinder v0.4 [79]. All single-copy protein orthogroups were selected for the phylogenomic tree reconstruction based on the software described above. The dendrogram generated was converted to an ultrametric tree with MEGAX software with a penalized likelihood method and a TN algorithm [80]. Calibration of the tree was performed using the divergence times of 15.78 My between *C. graminicola* and *C. sublineola* estimated previously [69]. The corresponding branch that clusters with *C. higginsianum* was used as an outgroup group. For running CAFE analysis, a lambda value (maximum likelihood value of the birth-death parameter) of 0.0395877 was assumed. The gene families with representative size variance were detected employing 1,000 random, number of threads = 10, and a *p-value* cutoff ≤ 0.01. The branches with a significant evolutionary value were identified based on the Viterbi algorithm with a *p-value* cutoff of 0.05. The analysis of expansions and contractions for each CYP family were obtained by using CAFE.

### Conservation of motif sequences analysis

Recognition and analysis of the motif sites in the multiprotein CYP sequences alignment obtained by MUSCLE v3.8.3 was performed using GeneDoc (http://nrbsc.org/gfx/genedoc). The residues assigned in the motif sites were reserved for analysis. Consensus logos corresponding to motif sequences were generated and visualized with the WebLogo webserver, plotting a stack of amino acids for each position [81, 82].

### Differential expression analysis of the *C. graminicola* M1.001 transcriptome

The expression profile analysis of the CYPome of *C. graminicola* during the host-pathogen interaction was carried out employing the *C. graminicola* M1.001 pathosystem expression data obtained by O’Connell et al. (2012). Illumina RNA sequencing data for three developmental stages were selected corresponding to the following conditions: 22 h post-inoculation- *in planta* appressoria (PA), 40 h post infection (hpi) - biotrophic phase (BP), and 60 hpi – necrotrophic phase (NP). Differentially Expressed *CYP* genes from the total set of Differentially Expressed Genes (DEGs) were selected for each pairwise comparison: NP vs PA, NP vs BP, BP vs PA. From Supplementary Table 14 of O’Connell et al. [14], we extracted normalized read counts. Differential expression analysis for each pairwise comparison was performed using the DESeq2 R package v.1.28.1 [83]. In this study, genes with a log2 fold-change ≥ 2 or ≤ -2, and a *p*-value < 0.05, were considered as DEGs, of which only the *CYP* genes were used in downstream analysis. Shared DEG *CYP*s were visualized by plotting a Venn Diagram generated with the Intervene web tool [84]. The R package pheatmap 1.0.12 was used for showing and comparing the number of each *CYP* family differentially expressed by the different infection stages [85]. Volcano plots were created with the following criteria: *p-value* 0.05 and ≤ log_2_foldchange□ = 2 by using the R package ggplot2 3.3.2 [86]. Intervene’s UpSetR module was used for preparing a general graphic UpSetR with all intersection of three pairwise comparisons. The DEG *CYP* gene information was used for recreating a schematic representation which infers the putative Secondary Metabolite Gene Clusters (SMGCs) expressed in the different infection stages.

### Structure-based dendrogram (SBD) construction of CYP enzymes of *C. graminicola* M1.001

The SBD was obtained according to the following: the hypothetical three-dimensional structures (TDS) belonging to the paralogous CYP’s corresponding to the families CYP65, CYP68, CYP504, CYP505, CYP526, CYP527, CYP530, CYP535, CYP552 and CYP570 were predicted for this analysis, by using Alphafold2 (AF) included in the ColabFold software [87, 88]. The modelling of TDS with AF was conducted employing 100 representative structures deposited in the Alphafold Database. The sequence alignments and template generations were generated using MMseqs2 and HHsearch included in ColabFold [89, 90]. The rankest TDS suggested by AF were selected for SBD reconstruction. The SBD was generated by using DALI software [91]. The dendrogram was visualized and edited with iTOL. Representative TDSs were visualized with Discovery Studio 2020 Client [92].

### Synteny analysis and assignment of the paralogous CYP genes to Secondary Metabolite Gene Clusters (SMGCs) in *C. graminicola* M1.001

AntiSMASH version1.2.2 [93], and the MycoCosm web portal (https://mycocosm.jgi.doe.gov/) were used to identify SMGCs. BLASTP was employed for manual classification of *CYP* genes into SMGCs. Synteny analysis was explored in 10 representative *CYP* families encoded in the genome of *C. graminicola*. The nearest neighbors clustered in the genomic context suggested for the Gene Tool deposited in NCBI were used for the analysis. The analysis of synteny among clusters was complemented also was performed with Clinker v.0.028 software [94].

### Supplementary Information

**Additional file 1: Table S1.** Number of P450 cytochromes harbored in representative species belonging to *Colletotrichum* genus.

**Additional file 2: Table S2.** Abundance of gene copies present in each CYP family in species belonging to *Colletotrichum* graminicola complex.

**Additional file 3: Table S3.** Number of shared CYP families among species belonging to *Colletotrichum* Graminicola s.c.

**Additional file 4: Table S4.** Quantitative values corresponding to expansions and contractions occurred during the evolution of the CYPome in species belonging to *Colletotrichum* Graminicola s.c. The analysis was performed by using CAFE version 4.2.1, employing a Viterbi algorithm with a *p*-value cutoff of 0.05.

**Additional file 5: Table S5.** Functional assignation of CYP families. Highlight colors represent the assignation of the function in each CYP family.

**Additional file 6: Table S6.** Gene gains and loss for each CYP family that have occurred during the evolution of the CYPome in species belonging to the Graminicola s.c. The analysis was performed using CAFE version 4.2.1.
